# Supporting collaborative use of the diabetes population risk tool (DPoRT) in health-related practice: a multiple case study research protocol

**DOI:** 10.1186/1748-5908-9-35

**Published:** 2014-03-21

**Authors:** Laura Rosella, Leslea Peirson, Catherine Bornbaum, Kathy Kotnowski, Michael Lebenbaum, Randy Fransoo, Patricia Martens, Patricia Caetano, Carla Ens, Charles Gardner, David Mowat

**Affiliations:** 1 Public Health Ontario, Santé publique Ontario, 480 University Avenue, Suite 300, Toronto, ON M5G 1V2, Canada; 2 Dalla Lana School of Public Health, University of Toronto, 155 College Street, 6th Floor, Toronto, ON M5T 3M7, Canada; 3 Institute for Clinical Evaluative Sciences (ICES), G1 06, 2075 Bayview Avenue, Toronto, ON M4N 3M5, Canada; 4 McMaster Evidence Review and Synthesis Centre, McMaster University Faculty of Health Sciences, School of Nursing, 1280 Main St. W., DTC Room 319, Hamilton, ON L8S 4K1, Canada; 5 Department of Community Health Sciences, Faculty of Medicine, University of Manitoba, Room S113–750 Bannatyne Avenue, Winnipeg, MB R3E 0W3, Canada; 6 Provincial Drugs Program, Manitoba Health, 300 Carlton Street, Winnipeg, MB R3B 3M9, Canada; 7 Simcoe Muskoka District Health Unit, 15 Sperling Avenue, Barrie, ON L4M 6K9, Canada; 8 Region of Peel Health Services, Peel Public Health, 7120 Hurontario Street, P.O. Box 667, RPO Streetsville Mississauga, ON L5M 2C2, Canada

**Keywords:** Diabetes population risk tool, Diabetes, Knowledge translation, Knowledge-to-action, Partnership, Knowledge broker, Public health, Risk tool, Chronic disease

## Abstract

**Background:**

Health policy makers have stated that diabetes prevention is a priority; however, the type, intensity, and target of interventions or policy changes that will achieve the greatest impact remains uncertain. In response to this uncertainty, the Diabetes Population Risk Tool (DPoRT) was developed and validated to estimate future diabetes risk based on routinely collected population data. To facilitate use of DPoRT, we partnered with regional and provincial health-related decision makers in Ontario and Manitoba, Canada. Primary objectives include: i) evaluate the effectiveness of partnerships between the research team and DPoRT users; ii) explore strategies that facilitate uptake and overcome barriers to DPoRT use; and iii) implement and evaluate the knowledge translation approach.

**Methods:**

This protocol reflects an integrated knowledge translation (IKT) approach and corresponds to the action phase of the Knowledge-to-Action (KtoA) framework. Our IKT approach includes: employing a knowledge brokering team to facilitate relationships with DPoRT users (objective 1); tailored training for DPoRT users; assessment of barriers and facilitators to DPoRT use; and customized dissemination strategies to present DPoRT outputs to decision maker audiences (objective 2). Finally, a utilization-focused evaluation will assess the effectiveness and impact of the proposed KtoA process for DPoRT application (objective 3). This research design utilizes a multiple case study approach. Units of analyses consist of two public health units, one provincial health organization, and one provincial knowledge dissemination team whereby we will connect with multiple regional health authorities. Evaluation will be based on analysis of both quantitative and qualitative data collected from passive (*e.g*., observer notes) and active (*e.g*., surveys and interviews) methods.

**Discussion:**

DPoRT offers an innovative way to make routinely collected population health data practical and meaningful for diabetes prevention planning and decision making. Importantly, we will evaluate the utility of the KtoA cycle for a novel purpose – the application of a tool. Additionally, we will evaluate this approach in multiple diverse settings, thus considering contextual factors. This research will offer insights into how knowledge translation strategies can support the use of population-based risk assessment tools to promote informed decision making in health-related settings.

## Background

Diabetes is rising at an alarming rate in Canada. In particular, Type 2 Diabetes Mellitus (T2DM) accounts for over 90% of diabetes and is a leading cause of death and disability [1]. Currently, 2.7 million Canadians are living with diabetes; this rate is expected to rise to 4.2 million by 2020 [2]. In Ontario, Canada’s largest province by population, the 2005 prevalence of diabetes exceeded the World Health Organization’s predictions for 2030 [3]. Similarly, diabetes prevalence has increased over time in the province of Manitoba, especially in the Northern region, which has a large proportion of Aboriginal residents [4]. There is growing concern that these trends may slow or even reverse life expectancy gains in developed countries [1].

Without urgent preventative action, diabetes will continue to deteriorate the health status of the Canadian population and overwhelmingly burden the healthcare system. Several governments and health policy makers at all levels have authoritatively stated that diabetes prevention is a priority through policy announcements and strategic system investments worldwide [5]; however, uncertainty exists regarding the specific type, intensity and target of interventions that will achieve the most effective and efficient impact overall and in priority populations.

Decision makers have access to self-reported prevalence data on diabetes, but rarely have access to diabetes incidence and true prevalence of physician diagnosed diabetes. They lack tools that facilitate the use of this data to inform population planning or prevention strategies. Specifically, they lack tools that can apply readily available data to determine optimal diabetes prevention strategies based on the unique socio-demographic and risk factor composition of populations of interest [6], including the optimal balance of population-based and high-risk strategies. Risk algorithms are used to estimate absolute risk of an outcome for an individual as a function of their baseline characteristics. In clinical medicine, risk algorithms are used for patient decision making and have contributed to important advances in individual treatment and disease prevention. However, risk prediction tools have rarely been applied outside of clinical settings at the individual level. Application to the population level has several benefits, including providing insight into the future burden of a disease in an entire region, examining the influence of specific risk factors on disease burden, exploring the potential health impact of various population-based and high-risk interventions, and providing information that can be used for health resource planning. In order for a tool to effectively inform population prevention strategies, it must address the needs of decision makers and provide relevant information to the health professionals and/or community stakeholders who will apply the results [7]. This includes addressing potential barriers, such as developing a tool that uses data readily available to knowledge users [8] (*i.e*., accessible to public health units and organizations). Other salient features that ensure a tool can be adapted include: practicality, applicability to important target populations, validity and robustness [9].

Considering the potential benefits of a population-based risk prediction tool and responding to the rising incidence and consequences of diabetes within Canada, the Diabetes Population Risk Tool (DPoRT) was developed and validated to estimate future diabetes risk based on routinely collected population data [10].

### The diabetes population risk tool (DPoRT)

DPoRT, developed by Rosella and colleagues [10], is a novel tool that offers an innovative way to make routinely collected population health data practical and meaningful for diabetes prevention planning and local decision making. The tool was designed to apply routinely collected and publicly available data, enabling its use by different users (*e.g*., epidemiologists, public health decision makers). To accomplish broad applicability, the tool works on risk factor data available to users at community, regional and provincial levels (*i.e*., Canadian Community Health Survey [CCHS]). A recent systematic review of all diabetes risk tools found that of over 90 existing tools, DPoRT was the only one built specifically to inform population intervention strategies [6]. DPoRT was also the only tool rigorously validated in two diverse external populations and across important subpopulations (*e.g*., ethnic groups) [11]. Specific uses of DPoRT include identifying at-risk groups, generating intervention scenarios that allow the user to weigh trade-offs of strategies, and estimating future number of diabetes cases and costs for the purpose of health resource planning. Despite these significant advantages, the uptake and utilization of DPoRT in health-related settings remains sparse.

### An integrated approach to KT for decision-support tools

Research has shown that passive knowledge translation (KT) strategies are likely insufficient to maximize uptake and utilization of knowledge products and innovations in various sectors [12-16], including public health [16]. In our experience, conventional researcher driven KT approaches [17] are insufficient for facilitating the use of tools such as DPoRT and the tailored knowledge products that result from such use. Furthermore, simply providing health-related organizations with DPoRT and letting them figure out how to use it will not effectively influence practice and decisions because of perceptions that the software must be run by the original developers. Since DPoRT and its proposed applications are novel, specific KT strategies to inform how to optimize its uptake and utilization are lacking. Nevertheless, interactive and integrated KT approaches have shown promise as a means to capitalize on the reach and potential of innovations [14,18-20], particularly when knowledge users are included as integral partners in the process [21-23]. Consequently, this study intends to facilitate the collaborative use and application of DPoRT by key decision makers and researchers through an integrated knowledge translation (IKT) approach.

We are not aware of any published studies of KT approaches specifically designed to support the application of tools designed to generate future population-level risk profiles to facilitate decision making. Work in related domains, including clinical decision-rules [24,25] and guidelines [26], demonstrates the importance of KT strategies that are tailored to both the specific properties of the tool as well as the intended users. In an effort to address this gap in the literature, we designed an IKT approach that involves partnering with regional and provincial health decision makers in two provinces to facilitate the uptake of DPoRT using several different strategies. The execution of this IKT approach is informed by the Knowledge-to-Action (KtoA) framework developed by Graham [15].

**Figure 1:**
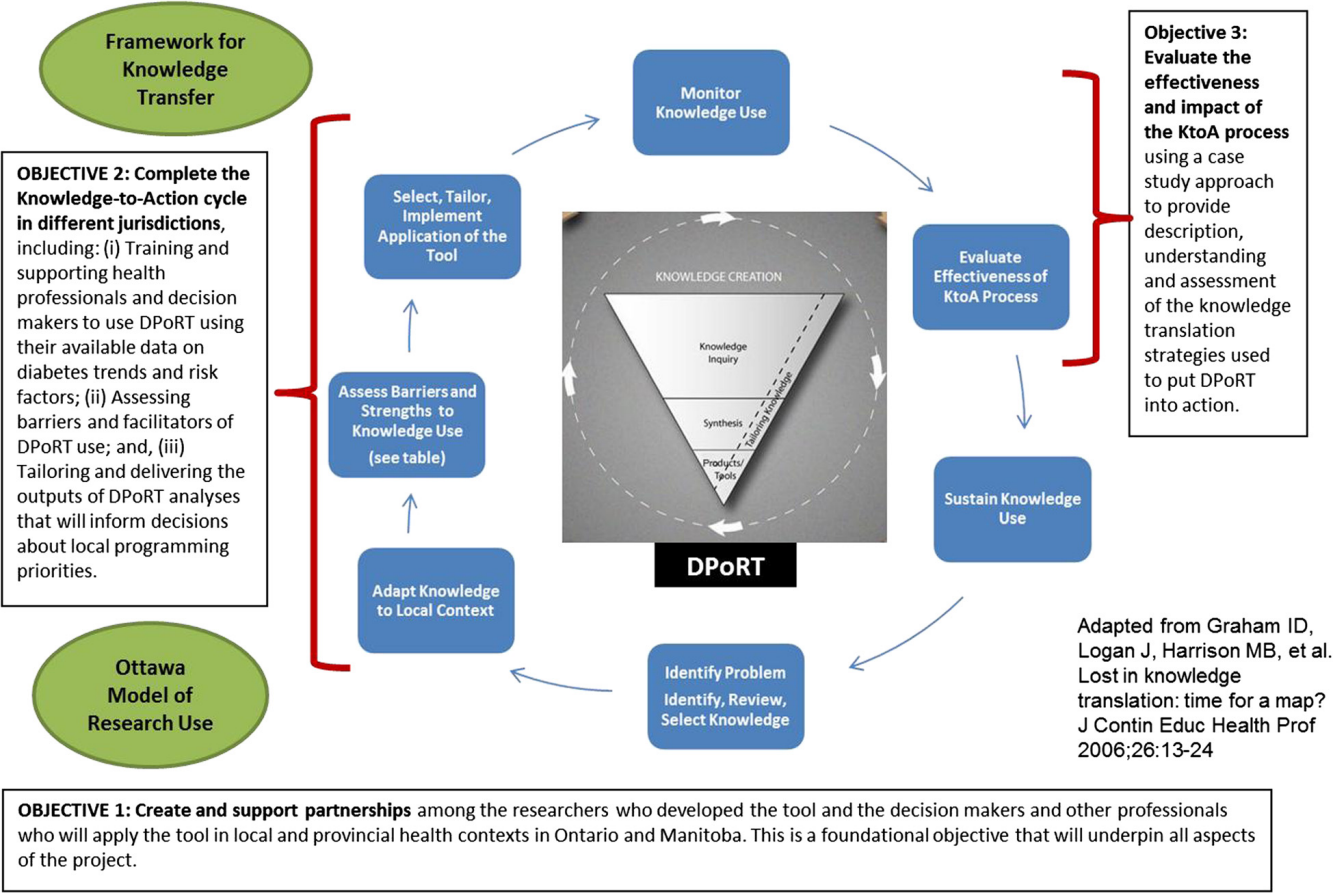
DPoRT adapted knowledge to action framework.

The KtoA framework describes the KT process as encompassing two key components, knowledge creation (represented by a funnel that distills and tailors information into useable knowledge products or tools) and action (facilitated through a dynamic context-driven process) [15]. The KtoA framework offers a suitable structure for conceptualizing past, present and future efforts to attain effective and contextually relevant applications of DPoRT. The knowledge creation phase was completed through the development, validation and refinement of DPoRT [10,11,27]. Furthermore, through pilot work with Peel Public Health in Ontario, we have confirmed that the current form of DPoRT is relevant for application. The action phase of the KtoA framework describes the actual tasks and activities required for implementation and uptake. However, research to inform the KT and application of population-level risk prediction tools (*e.g*., DPoRT) by knowledge users in health-related settings (*i.e*., the KtoA action cycle) has yet to be undertaken. Thus, this protocol addresses the process of moving DPoRT into action through the collaborative efforts of researchers and DPoRT users. Our conceptualization of the KtoA framework, adapted for this project, is presented in Figure 1. The details of the conceptual map guiding this work are forthcoming in a separate manuscript.

### Goals and objectives

The overarching goal of this project is for researchers and decision makers in varied health-related settings to work collaboratively to build capacity and facilitate the use of DPoRT as a strategic aid for population-based risk assessment, intervention and planning decisions. In an effort to empower health organizations to use DPoRT for their own decision making and surveillance activities, we have developed an IKT strategy in partnership with regional and provincial decision makers in Ontario and Manitoba, Canada. To achieve this goal, the specific objectives of this project are to:

1. Create and support partnerships with DPoRT users in health-related settings, through use of a knowledge brokering (KB) team. This is a foundational objective that will underpin all aspects of the project. In particular, the KB team will: facilitate and support ongoing relationships with DPoRT users; provide tailored training to help health setting staff learn to use DPoRT; and adapt DPoRT application to the local sites’ needs

2. Complete the action cycle of the KtoA framework [15] in multiple settings, including: train and support health professionals and decision makers to use DPoRT with their available data on diabetes trends and risk factors; assess barriers and facilitators of DPoRT use; and tailor and deliver the outputs of DPoRT to inform programming and policy decisions.

3. Evaluate the effectiveness and impact of the KtoA process using a case study approach to provide description, understanding and assessment of the KT strategies used to put DPoRT into action. Our specific evaluation objectives are: to evaluate the effectiveness of the partnerships; to evaluate the effectiveness of the KtoA approach; and to identify strategies that facilitate uptake and overcome barriers to DPoRT use, including core elements needed for sustained use.

## Methods/Design

### Overview

Health organizations in this study and the populations they serve vary considerably, and there is increasing recognition that differences in context make it difficult or impossible to replicate interventions exactly the same way across different conditions [28]. It has been suggested that we should continue to expect standardization in the process, sequence and function of an intervention, but that we allow for adaptation to the dynamic and unique properties of each context [15,22,29-33]. With this advice in mind, we have outlined a set of activities that we believe must be carried out in each setting to ensure valid, reliable and useful application of DPoRT. However, as we enter into this process and at the outset of each step, key stakeholders will be invited to engage in exchanges and assessments that will help tailor the form of these activities to suit their unique needs and realities.

To facilitate our intended IKT approach, we will use a KB team. In the literature, knowledge brokers have been described as trusted, knowledgeable, skilled and solution-oriented individuals or groups who act as ‘go-betweens’ to bring key stakeholders together in formal and informal venues for the purpose of acquiring, sharing and applying knowledge to improve decision making and/or the management/delivery of services [34-39]. Knowledge brokering is becoming a popular strategy for linking the producers and users of knowledge to facilitate the reach and effective uptake of innovative research. There is an expanding literature describing the effectiveness of initiatives that have utilized knowledge brokering approaches (*e.g*., public health [40], mental health [41], community-based non-profit organizations [42], and clinical practice [43,44]). As supported by other experiences with knowledge brokering in public health [33], we believe this is an appropriate approach to adopt as our primary KT strategy to support all project objectives. However, in contrast to strategies that use a single knowledge broker, we have established a KB team that spans the content expertise areas required to support the uptake and use of DPoRT. Specifically, the KB team consists of: a member of the DPoRT development research team, an epidemiologist trained in using DPoRT, and two research coordinators with expertise in KT and evaluation in health-related settings. The KB team will: provide support in building the partnerships (objective 1), lead, coordinate and monitor training and uptake activities, help to navigate and appraise DPoRT applications, identify barriers, and encourage collaborative, creative and context-specific problem-solving (objective 2). In addition, members of the KB team will facilitate and collect data for the evaluation (objective 3). DPoRT computations are relatively straightforward, but application of the tool may be challenging for the users, and one-off strategies such as a training workshop on how to run the software will not be sufficient to realize the potential value of the tool for informing decision making. The strategy must also consider that some of the users will not be applying DPoRT themselves but rather using the outputs of DPoRT. We believe that sustained partnerships between DPoRT users and the KB team will facilitate DPoRT application and ensure effective uptake and use.

We have designed a utilization-focused evaluation [45,46] leveraging the collaborative relationship with the DPoRT users. Our approach reinforces the IKT nature of this project such that the KB team facilitating the IKT approach will also be actively engaged in evaluation activities [47], and the DPoRT users will be involved in focusing the evaluation [46]. The evaluation will employ a multiple case study approach [48] with the participating organizations as the units of analyses. The evaluation will occur throughout the action cycle and will be based on analysis of both quantitative and qualitative data collected from passive (observer notes) and active (surveys/interview) methods. This approach is particularly useful for this project because it can be applied when the activities involve complex social interactions, when control over variables is limited, and when the boundaries between the phenomenon under study (DPoRT use) and the application contexts (varied health-related organizations) are blurred [48,49]. Case study design also allows for an in-depth understanding of the action cycle in each setting, to explore organizational capacity, and to identify the barriers and facilitators to DPoRT use. Within-method triangulation [50] will be used to gather in-depth information at multiple points in time and from multiple sources.

### Ethics approval

The Research Ethics Board at Public Health Ontario (PHO) has approved this study protocol.

### Sample

When DPoRT was developed, epidemiologists and decision makers (*e.g*., managers, directors, Medical Officers of Health, Chief Executive Officer or equivalent) working in health-related organizations were identified as the ideal users of the tool. It was expected that they would benefit most from identifying risks based on their unique community structures that could be used to directly inform jurisdictional programming decisions. As elaborated below, the participating organizations represent diverse contexts and will permit exploration of how the action cycle of the KtoA framework plays out under different conditions as well as allow us to test the robustness of our IKT approach.

### Ontario

The two participating public health units in Ontario have different internal capacities and serve different types of communities. Peel Public Health (PPH) is a local public health department serving over 1.3 million residents in three primarily urban municipalities. PPH is part of the Health Services Department within the regional government whereby Peel Regional Council serves as its Board of Health. PPH’s 700-member staff provide services specified in the Ontario Public Health Standards and the Health Protection and Promotion Act [51]. Peel is a rapidly growing and highly diverse community with a large immigrant population (49%). Within PPH there will be one main group of DPoRT users: the epidemiology team, which provides synthesized health status data to public health teams to support informed decisions about programming. We conducted pilot work in Peel prior to developing this protocol, which informed our IKT approach as well as training materials.

Simcoe Muskoka District Health Unit (SMDHU) is a local public health unit situated at the north end of the Greater Golden Horseshoe and in the heart of Ontario’s ‘Cottage County’. The health unit’s jurisdiction covers over 8,000 km^2^ and includes 2 upper tier governments, 2 cities and 24 smaller municipalities. In 2006, the total population for Simcoe and Muskoka was 479,767; projections suggest this number will be 40% higher and will include many seniors by 2025. The SMDHU Board of Health is an autonomous governing structure made up of appointees from the County of Simcoe, District of Muskoka, City of Barrie, City of Orillia and Provincial designates. The health unit has 400 staff members. Within SMDHU there will be two main beneficiaries of DPoRT: the Planning and Evaluation team within Corporate Services and the Chronic Disease Prevention Healthy Lifestyle (CDP-HL) team within Healthy Living Services. The Medical Officer of Health, one epidemiologist, and one manager from the CDP-HL team will be the primary participants.

### Manitoba

DPoRT was validated in Manitoba because of the different population structure from Ontario, thus demonstrating the robustness of the tool [10]. More importantly, diabetes is a significant health problem in Manitoba, and diabetes prevention and planning is a priority for decision makers. We will engage with several decision makers in Manitoba representing a range of populations, as well as a diverse set of DPoRT users responsible for diabetes prevention and planning. Manitoba Health is a department within the Government of Manitoba which operates under the provisions of the legislation and the responsibilities of the Minister of Health. The legislation, as well as emerging health and healthcare issues, guides the planning and delivery of healthcare services for Manitobans. Manitoba Health is currently undertaking a Chronic Disease Prevention Initiative (CDPI), which will benefit from DPoRT application to inform the most relevant strategies for diabetes prevention. Regional Health Authorities (RHAs) via ‘The Need To Know’ (NTK) Team: We will apply DPoRT to the local regions in Manitoba via the NTK Team, which is a well-established conduit for KtoA in the province [18,52,53]. The three objectives of the NTK Team are to create new knowledge of high relevance to policy and planning imperatives in the regions, to engage in capacity building activities among the partners, and to ensure that the research is disseminated and applied at the regional level. The NTK Team involves scientists from the Manitoba Centre for Health Policy (MCHP), high-level planners from each of Manitoba’s five RHAs and Manitoba Health. The team meets three times a year, for two-day meetings. Team members take on the critical role of knowledge uptake when they facilitate roundtable discussions in the annual MCHP/RHA Workshop Days, where RHA board members, chief executive officers, vice presidents, program managers, Medical Officers of Health, and others examine current MCHP research report findings. These workshops are designed specifically so that RHAs can learn about better ways to use population-based data to guide priorities for regional strategic plans and operations. Disseminating DPoRT to the RHAs via the NTK team is a resourceful way of leveraging an existing and effective KT conduit. Through the NTK team, interested RHAs will be connected to the KB team and invited to participate in the KtoA process. In order to build capacity for DPoRT use, Manitoba Health will work in collaboration with the interested RHAs.

## Data sources

### Observational notes

Throughout the KtoA cycle, the KB team will keep a record of the nature and sequence of events occurring at each site. At an initial site meeting and throughout the KtoA cycle, the KB team will inform participating staff about the collection of observational notes, the type of data that will be logged, the reason for recording such notes, and will explain that individuals can opt out if they prefer to not have their interactions with the KB team logged. Potential events that may be recorded include: requests by DPoRT users for technical support or resources related to DPoRT, discussions with the KB team to troubleshoot barriers to DPoRT use, discussions to support the development of knowledge products, etc. The log will record the type of interaction that the KB team engages in (*e.g*., phone, email, in-person meeting), role of the individual(s) spoken with, type of activity being conducted (*e.g*., facilitating, preparing, checking-in), length of time spent on interaction/activity, purpose of the interaction, actions taken to address issues/concerns, and outcomes/decisions that followed. The KB team will also keep a reflexive journal of critical reflections on partner interactions and setting processes. The purpose of recording observational notes is to understand the effectiveness and intensity of the knowledge brokering strategy as well as provide feedback on how the KB team can improve, given that we are utilizing an IKT approach.

### Post-training survey/After action review

The KB team will build capacity in using DPoRT at each site by delivering a training workshop. Each organization will determine who will attend training based on their anticipated use of DPoRT; these details will be discussed during initial site meetings. Additional training activities, such as one-on-one support, may be provided. To evaluate the effectiveness of the training activities, the KB team will administer a survey and After Action Review (AAR) [54] to individuals who participated in the DPoRT training workshop. The brief post-training survey will take approximately five minutes to complete, and will be used to assess the effectiveness of training delivery and level of confidence and comfort with using DPoRT independently. The AAR will be completed within two to four weeks of the training and will consist of a facilitated half-hour discussion to gain more details about experiences with the DPoRT training, and to identify aspects that went well or need improvement.

### Partnership self-assessment tool

In order to assess the effectiveness of the partnership between the KB team and each site, participants will be invited to complete the Partnership Self-Assessment Tool (PSAT) developed by the Center for the Advancement of Collaborative Strategies in Health [55] based on the Partnership Synergy Framework [56]. PSAT measures team synergy relative to: leadership, efficiency, administration, management, and sufficiency of resources. PSAT is a commonly used and validated measure for assessing the effectiveness of a partnership, which is a critical dimension of our IKT approach. In line with recommendations from the PSAT developers, this measure will be administered approximately six months after the commencement of the partnerships with each DPoRT user site.

### Semi-structured interviews

Approximately two to three months after DPoRT users have begun using the tool for their site-specific purposes (*e.g*., surveillance, program planning, etc.), a member of the KB team will conduct semi-structured interviews at each site with those who have used DPoRT (either directly or indirectly via outputs). The purpose of these interviews is to understand the effectiveness and impact of the KtoA process on DPoRT uptake, the effectiveness of the partnerships, and to assess actions, such as training and support, taken by the KB team. The questions in the interview guide will follow the OMRU guidelines [55] and probe elements of the KtoA process cycle [19].

### Data coding and analysis

Qualitative analysis will be conducted to identify emerging themes that require further exploration and to inform/or redirect the ongoing KtoA process. To inform the organization and coding of the qualitative data, we will a priori identify sensitizing concepts [48,50] structured as coding guides from the analytic frameworks guiding this project [14,15,56,57]. In-depth descriptions will be created for each setting that are holistic and context sensitive [48] using descriptive and iterative methods [58]. Additional coding domains will be developed as needed. All coding will be carried out by one individual; a sub-sample of the data will be coded by two independent researchers in order to assess confirmability through analyst triangulation [54]. We will use NVivo9 as a qualitative data management and analysis tool. Quantitative data derived from user surveys and user metrics will be analyzed and managed using SAS statistical software and will include descriptive summaries (such as means, frequencies) and, where applicable, correlations and bivariate statistics.

### Limitations

Since DPoRT users will consist of employees at health-related organizations, some participants may not feel comfortable revealing sensitive information related to their experiences with DPoRT, the training workshop, and the ongoing KB team support. In an effort to enhance participant confidence, letters of information will be provided to explain the measures that the KB team will take to protect confidentiality. For example, identifiable information will be stripped from transcripts, and interviews will be conducted in private rooms. Surveys and observational notes will not record identifiable information.

Additionally, given that implementation process-related data will be collected several months following the establishment of partnerships and training workshops, recall bias may limit the accuracy of DPoRT users’ descriptions of the implementation process. However, the use of multiple sites, and data sources (*i.e.,* triangulation) should mitigate the potential for recall bias [50].

## Discussion

DPoRT can be used to inform diabetes prevention strategies and support local decision making and planning. In this protocol, we propose a novel IKT approach to partner with local and provincial public health decision makers in two provinces to facilitate the uptake of this innovation. This project directly addresses the application of a novel decision-support tool and evaluates the effectiveness of this adapted KtoA process in multiple health-related settings in Canada. As a result of this project, several health organizations will have the capacity to run DPoRT and incorporate it into their surveillance activities and to forecast the effects of prevention for both health systems (forecasting future medical needs and expenses) and health planning (forecasting future disease burden and estimates of the effects of diabetes prevention). This project features a unique modification of the conventional KB strategy (a KB team approach), which will be examined as part of the evaluation. Our novel application of the KtoA framework (the application of a tool versus previously generated knowledge), and our evaluation of this strategy will be useful to others designing or evaluating KT strategies, particularly on the application of decision support tools, which are gaining popularity. The ultimate goal of DPoRT utilization would be to impact health outcomes, specifically diabetes incidence, as well as the consequences of diabetes, including cardiovascular disease, stroke, kidney disease, etc. Both diabetes and its consequent comorbidities may be reduced by the provision of more effective and efficient programs informed by DPoRT outputs. Importantly, this research will offer insights and lessons learned about the contemporary phenomenon of KT strategies to support uptake and application of population-based risk assessment tools for informing decision making regarding prevention strategies that can be used by other health-related settings interested in pursuing similar efforts.

## Abbreviations

AAR: After action review; CCHS: Canadian community health survey; CDP-HL: Chronic disease prevention – healthy living; CDPI: Chronic disease prevention initiative; DPoRT: Diabetes population risk tool; KB team: Knowledge brokering team; KtoA: Knowledge to action; KT: Knowledge translation; IKT: Integrated knowledge translation; MCPH: Manitoba centre for health policy; NTK: Need to know (team); OMRU: Ottawa model of research use; PHO: Public health Ontario; PSAT: Partnership self-assessment tool; RHA: Regional health authority.

## Competing interests

The authors declare that they have no competing interests.

## Authors’ contributions

This manuscript was prepared based on a Canadian Institutes of Health Research funded grant developed by LR, LP, DM, CG, PC, RF and PM. The grant was adapted into a draft manuscript by LR, CB, and KK. All authors contributed to the refinement of the protocol and have approved the final version of this manuscript.
